# Bulk effect of the deltoid muscle on the glenohumeral joint

**DOI:** 10.1186/s40634-014-0014-9

**Published:** 2014-11-04

**Authors:** Claudio Rosso, Andreas M Mueller, Brett McKenzie, Vahid Entezari, Andrea Cereatti, Ugo Della Croce, Arun J Ramappa, Ara Nazarian, Joseph P DeAngelis

**Affiliations:** Center for Advanced Orthopaedic Studies, Beth Israel Deaconess Medical Center and Harvard Medical School, Boston, MA USA; Orthopaedic Department, University Hospital Basel and University of Basel, Basel, Switzerland; Department of Orthopaedic Surgery, Beth Israel Deaconess Medical Center, Harvard Medical School, 330 Brookline Avenue, Stoneman 10, Boston, MA 02215 USA; Information Engineering Unit, POLCOMING Department, University of Sassari, Sassari, Italy; Altius Swiss Sportsmed Center, Rheinfelden, Switzerland

**Keywords:** Bulk effect, Shoulder, Biomechanics, Pitching, Deltoid muscle, Motion analysis

## Abstract

**Background:**

There remains controversy on the role of the deltoid on glenohumeral translations during basic and pitching motions. We thus studied the passive effect of the deltoid on the deltoid glenohumeral joint center (GHJC).

**Methods:**

Six shoulders were tested using an automated mechanical system. A baseline motion pattern of the intact specimen was contrasted with glenohumeral translation after removal of the deltoid. Each condition was evaluated in abduction (ABD) and an abbreviated throwing motion (ATM) using retro-reflective, bone-embedded markers. The absolute trajectory and the area under the curve (AUC) for each motion were calculated and glenohumeral kinematics with respect to the GH translation were compared.

**Results:**

The removal of the deltoid resulted in significant changes of the GH translation. During 30-60° of ABD, it resulted in a superior and more anterior GH translation, while in the 60-90° segment in a more inferior and medial GH translation. During 90-120°, the GH translation was medialized. In the pitching motion from maximum external rotation to 90° of external rotation (ER), the removal of the deltoid resulted in a more superior, anterior and lateral GH translation. Thus limits anterior translation in the abduction-external rotation position. In the remaining segments (90-80° and 80-45° of ER), it resulted in a lateralization of the GH translation.

**Conclusions:**

Modelling the throwing shoulder, the deltoid has a significant influence on glenohumeral motion. Athletes with deltoid dysfunction and limited range of motion are at risk for injury due to the resulting change in their throwing mechanics.

## Background

The shoulder has the greatest range of motion of all joints in the human body. It benefits from mobility at the glenohumeral, sternoclavicular, acromioclavicular joints and scapulothoracic articulation and functions to position the hand in space (An et al. 1991). However, since the glenohumeral joint (GH) has little bony stability, its supporting passive (labral and ligamentous) and dynamic (muscular) restraints help to preserve shoulder function.

While a number of muscles are central to the function of the shoulder in *in-vivo* settings, the deltoid together with the supraspinatus muscle have a significant role in initiating abduction and flexion when the arm is at rest (Reed et al. 2013). The deltoid muscle makes up approximately 20% of the shoulder muscles (Bassett et al. 1990; Lee and An 2002), where its most important function is humeral elevation due to its largest moment arm in comparison to all shoulder muscles (Kuechle et al. 1997). Additionally, in patients with rotator cuff arthropathy, the deltoid serves as the primary source of arm elevation. In this condition, the three main segments of the deltoid (anterior, middle and posterior) are crucial, such that the success of a reverse total shoulder arthroplasty hinges on the health and function of the deltoid (Ackland et al. 2010, 2011; Gulotta et al. 2012). Moreover, the deltoid muscle has been shown to passively affect the superior-inferior translation of the humeral head and limit anterior glenohumeral translation when the arm is abducted and externally rotated and thus is said to contribute to glenohumeral stability (Halder et al. 2001; Kido et al. 2003).

In passive *ex-vivo* studies, the deltoid has extensively been studied in conjunction with reverse shoulder arthroplasty but not in the intact joint (Ackland et al. 2011; Henninger et al. 2012). It has been proposed that its muscle bulk creates pressure that may increase joint stability and thus decrease glenohumeral translation (“bulk effect”) (Ovesen and Nielsen 1986; Ackland et al. 2011; Kido et al. 2003). The “bulk effect” has never been clearly defined but it is commonly accepted that the passive weight of the deltoid could be considered as the “bulk effect”. As a result of its superficial position, the deltoid may have an important role as a passive restraint (Howell and Galinat 1988). Both, Colachis et al. and Markhede et al. found that loss of function of the deltoid did not increase glenohumeral translation (Colachis et al. 1969; Markhede et al. 1985). Colachis et al. rendered the deltoid muscle inactive using an axillary nerve block, while Markhede et al. assessed the remaining function of the upper limb after removal of the deltoid muscle following tumor resection. While the removal of the deltoid muscle resulted in a decrease in strength, the loss was less than predicted with *a priori* calculations.

### However, there is no universally accepted dogma

We thus hypothesized that the *removal of the deltoid muscle would not affect glenohumeral translation* in our validated model (Rosso et al. 2013; Entezari et al. 2012; Mueller et al. 2014). The aim of the study was to prove this.

## Methods

### Testing apparatus

**Figure 1:**
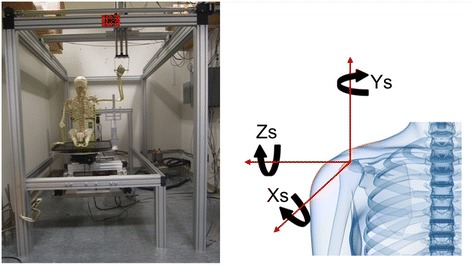
**Left side: Testing apparatus with a lower (small) frame and an upper (large) frame.** The upper frame can move in X, Y and Z directions, while the lower frame can move in X, Y, Z directions and rotate around the Y axis. Right side: An illustration of the system axes according to the ISB (International Society of Biomechanics) standards. The Z axis represents the medio-lateral axis while Y represents the supero-inferior axis and the X axis the antero-posterior axis. (Wu et al. 2005).

Our validated and published robotic testing system that generates automated motion segments for a cadaveric torso over a designated trajectory was used in this study (Rosso et al. 2013; Entezari et al. 2012). The robotic system consists of lower (torso) and an upper (hand) frames that provide linear as well as rotational motion along seven axes. The lower frame generates motion along X, Y, Z axes and around the Z axis, while the upper frame generates motion along the X, Y and Z axes, as highlighted in Figure 1. Motion was generated using linear and rotary closed loop actuators that are controlled via a centralized programmable system to generate any motion trajectory within the actuators’ limits. Limits and home switches were combined with encoders to produce closed loop feedback for each axis, ensuring safety for the cadaver/operator and precision. A detailed description of the apparatus has recently been published (Entezari et al. 2012). Also, the precision and accuracy of the testing system in reproducing pure and complex trajectories has been established in a separate publication (Rosso et al. 2013). Torsos were mounted onto a rod fixture while held in place with volume expanding foam and were mounted onto the lower frame. The end effector of the upper frame was secured to the radius and the ulna using a Schanz pin.

### Cadavers and motion analysis

Experiments were carried out on six fresh-frozen human torsos acquired from Medcure Anatomical Tissue Bank (Orlando, FL, USA). All specimens had intact shoulders with no history of shoulder pain. During dissection, we found all rotator cuffs to be intact while the status of the long head of the biceps could not be evaluated as the rotator cuff was not removed. More demographic data can be found in Table 1.

**Table 1:** **Demographic data**

**Age**	**Cause of death**	**Height [m]**	**Weight [kg]**	**BMI**	**Gender**	**Race**
64	Lung Cancer w/mets	1.88	125	35.3	m	Caucasian
57	Respiratory Failure	1.88	139	39.4	m	Caucasian
60	Esophageal Cancer w/Mets	1.93	109	29.2	m	Caucasian
64	Prostate Cancer	1.78	61	19.4	m	Caucasian
50	Myocardial Infarction	1.70	64	21.9	m	Caucasian
49	Glioblastoma	1.78	95	30.1	m	Caucasian
67	Myocardial Infarction	1.85	84	24.4	m	Caucasian

Motion data, consisting of three dimensional positions of reflecting markers in the global coordinate system, were acquired using a five-camera stereo-photogrammetric system (ProReflex Cameras®, Qualisys, Gothenburg, Sweden, 120 frames/s). The acquisition volume was a 1.5-m-sided cube. This system has been validated and can resolve differences in glenohumeral translations as little as 0.5 mm (Rosso et al. 2013; Entezari et al. 2012).

**Figure 2:**
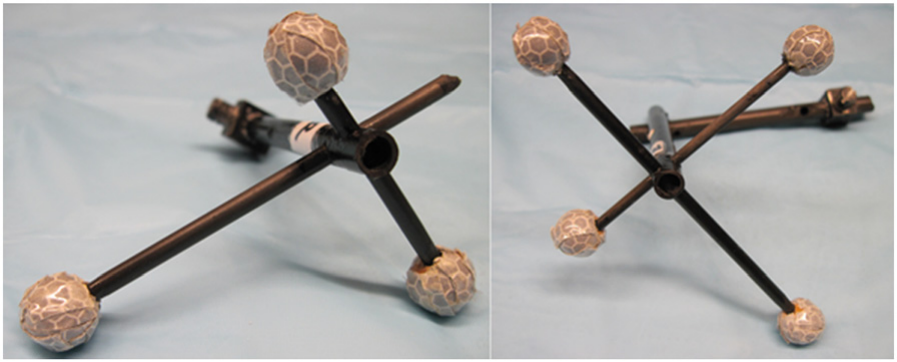
**Marker cluster.** This figure depicts the four marker cluster used.

While the specimen was secured to the robotic system, a steel pin equipped with a four marker cluster (transosseous bi-cortical) was implanted into the humeral diaphysis (Figure 2). An acromion marker cluster, equipped with four markers was directly attached to the acromion using three screws (4 mm × 10 mm). The acromion marker cluster was made of an alloy triangular base specifically shaped to be positioned over the flat part of the acromion (Cereatti et al. 2014). Before starting the dynamic acquisitions, with the arm hanging along the side of the torso, the positions of the following anatomical landmarks [28] were measured and registered with respect to the relevant bone marker clusters using a pointer equipped with a four-marker cluster according to the Calibration Anatomical System Techniques (CAST) [22]: the most caudal point on lateral epicondyle (LE), the most caudal point on medial epicondyle (ME), the trigonum spinae scapulae (TS), the angulus inferior (AI), the angulus acromialis (AA), the most ventral point of processus (PC) and the most dorsal point on the acromioclavicular joint (AC).

The poses of the marker cluster coordinate system of the humerus and the scapula, relative to the global coordinate system, were estimated using a SVD (singular value decomposition) technique (Soderkvist and Wedin 1993). The anatomical coordinate systems of humerus and scapula were defined according to the ISB (International Society of Biomechanics) recommendations (Figure 1) [21] and were registered relative to the corresponding marker cluster coordinate systems according to [22].

The position of the glenohumeral joint center was determined according to the regressive equation described by Meskers et al. (Meskers et al. 1998). In particular, while the arm was hanging along the side of the torso, this position with respect to the scapula marker cluster was identified as the baseline neutral position. The position was then expressed with respect to this neutral position. This procedure allowed to define two glenohumeral joint center positions for the scapula and humerus, respectively. These two positions were assumed to coincide in the reference shoulder position (arm hanging along the side of the torso).

In order to recreate every day and sport-specific motions, two different types of motion segments were chosen for the study: 1) humeral elevation in the coronal plane (abduction, ABD) from 30° to 150° (120° range) and 2) abbreviated throwing motion (ATM). According to the definition of the baseball pitching motion (Meister 2000), we defined an abbreviated throwing motion ranging from the late cocking (maximal external rotation of the humerus) to the deceleration phase. The ATM motion was tested from maximal external rotation in 90° of abduction of the humerus (abduction- external rotation (ABER) position) into approximately 45° of external rotation (75° range). This would also recreate the abduction-external rotation moment as published by Kido et al. (Kido et al. 2003).

Abduction was chosen as a basic motion of the arm, and the abbreviated throwing motion was chosen as a uniquely human motion (Roach et al. 2013), where maximal external rotation removes laxity from passive restraints, thereby further increasing motion repeatability.

**Figure 3:**
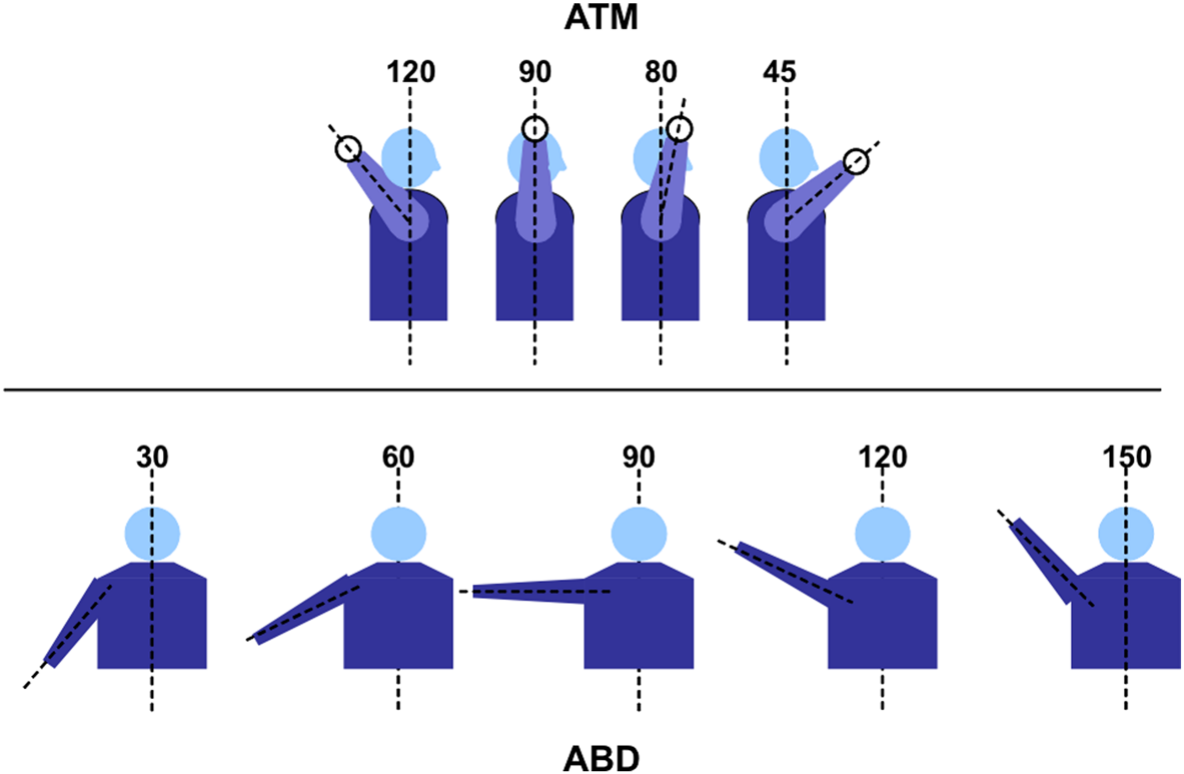
**Illustration of the range of motion of the abbreviated throwing motion (ATM, top) and abduction (ABD, bottom).** The ATM segments were defined according to the phases of the pitching motion as published by Meister.(Meister 2000): Segment I ranged from maximal external rotation at 90° of humeral abduction to 90° of external rotation, segment II ranged from 90° or ER to 80° of external rotation of the humerus, and segment III ranged from 80° of external rotation to 45° of external rotation and was at the same time the end of the motion (end of acceleration phase). The abduction was segmented into 30-60-90-120-150 degrees of abduction.

**Figure 4:**
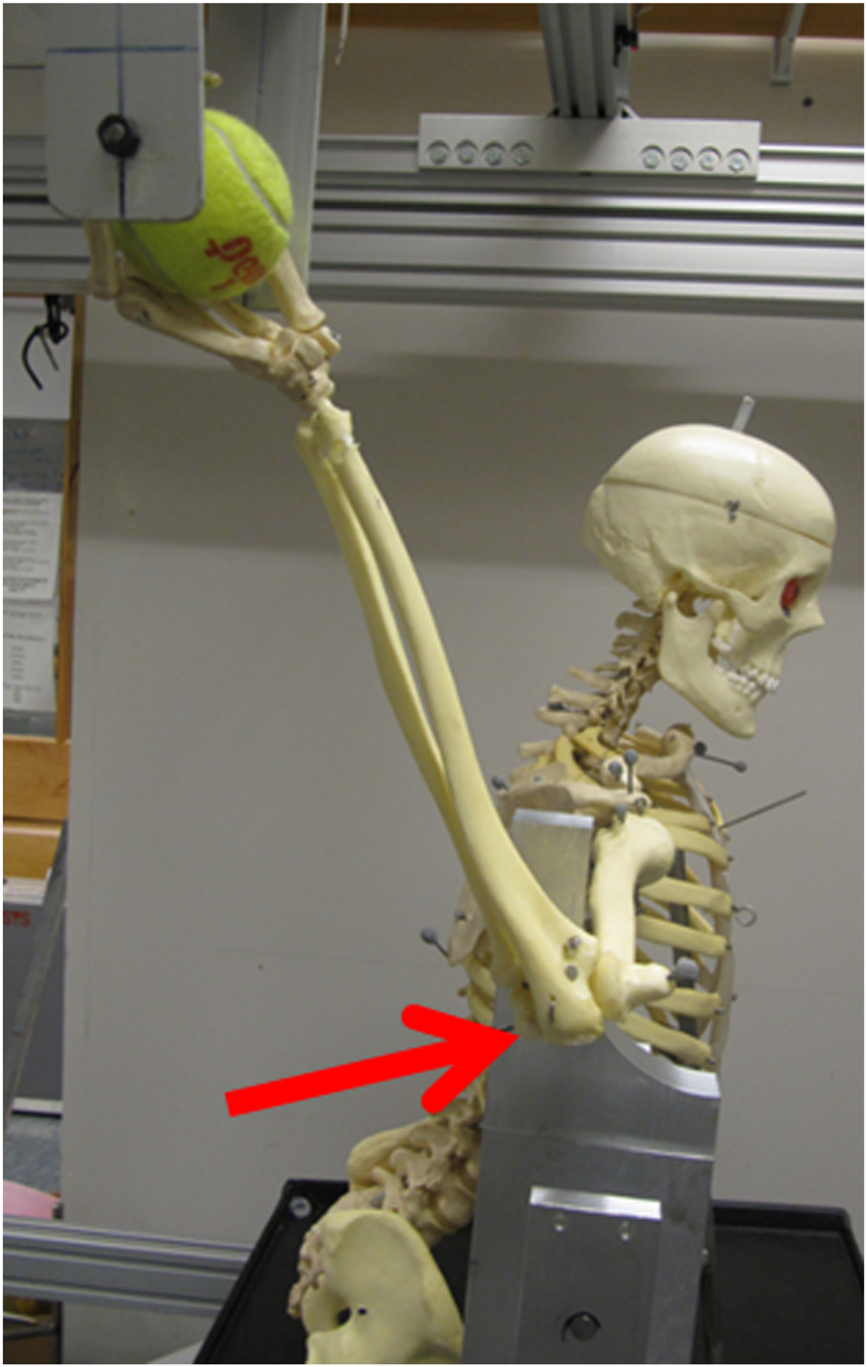
**The posterior restraint (red arrow) permits the humerus to externally rotate, while the hand is pulled dorsally.** This was used for the abbreviated throwing motion (ATM) for creating the external rotation needed for replicating the late cocking phase of baseball pitching.

Figure 3 depicts the range of each motion segment, while Figure 4 depicts the posterior restraint needed to attain maximal external rotation in the abbreviated throwing motion. For each of the abovementioned trajectories (ABD and ATM), two shoulder conditions were tested in triplicates: 1) cadaver with deltoid on (DON), and 2) cadaver with deltoid off (DOFF). For the DON configuration, only the skin was removed prior to data acquisition. Prior to DOFF data acquisition, the entire deltoid muscle was identified and dissected exposing the subacromial space and rotator cuff. The remaining tissue was kept moist with physiologic 0.9% saline throughout the experiment, and no other restraints were introduced into any anatomic locations (i.e. no rigid fixation of scapula). External rotation of the arm during the ATM motion was ensured by using a posterior restraint to the humerus. The posterior restraint had to be implemented as else a posterior motion of the arm would result in horizontal abduction and not in external rotation of the humerus (Figure 4). The posterior restraint was applied to the humerus while the robotic device moved posteriorly. In this way, the arm was forced into external rotation (Figure 4). As the calculation of the removal of the deltoid was done in comparison to the motion with deltoid (DON) using the same posterior restraint, its effect can be neglected. Testing was conducted with no resting time between each repetition.

A total of 12 (2 conditions × 2 shoulder motion × 3 trials) data sets were acquired for each specimen, and for each trial at each instant of time, the position vectors of the humerus GHJC were expressed in the scapular reference system. Glenohumeral translation was defined as the displacement vector of the humerus GHJC with respect to its position at the beginning of the motion under analysis expressed in the scapula anatomical frame.

### Data analysis

The absolute trajectory and the area under the curve (AUC) for each specimen at each motion segment were generated. AUC was calculated for each condition on each axis using the trapezoidal rule in order to appropriately assess the path-dependent motion (Matlab version 12, Mathworks, Natick, MA, USA). Given the real time data acquisition capability of the testing system used in this study, the motion trajectory itself could be compared between different testing conditions. To that end, the area under the curve of the motion trajectory was used as a quantitative surrogate for variations in the motion path across different conditions. Area under the curve, averaged across the three trials per condition, was used to compare differences in the motion paths between different conditions (DON and DOFF) for each motion segment and each motion (ABD and ATM).

Motion segments were divided into discrete steps to facilitate statistical comparison of data between different conditions (DON and DOFF). The ABD motion was discretized into five 30° increments (ABD^30°^^, 60°^^, 90°^^, 120°^^, 150°^). Similarly, the ATM motion was discretized into 4 increments to match pitching phases ABER^MAX^ (maximum external rotation in 90° of abduction, early cocking), ABER^90°^ (mid acceleration), ABER^80°^ and ABER^45°^ (ball release). In the ATM motion, the arm was at 90° of abduction and pivoted around the humeral axis. Repeated measures Analysis of Variance (ANOVA) with arm angle and test repetition (3 per specimen per condition) as within-subject factors and group as between-subject factor was conducted for X, Y and Z translations. For cases were the assumption of sphericity was not met, the Huynh-Feldt correction was used to adjust the degrees of freedom of the F test. The Wilcoxon Signed–Rank test was used to compare the areas under the curves (AUC) between the conditions. The AUC comparisons were conducted for the entire curve and for each curve segment between arm angles (Figure 3. ABD: ABD^30°^^−60°^, ABD^60°^^−90°^, ABD^90°^^−120°^, ABD^120°^^−150°^ and ABD^30°^^−150°^ and ATM: ABER^MAX-90°^, ABER^90°^^−80°^, ABER^80°^^−45°^ and ABER^MAX-45°^). Data analysis was performed using SPSS (version 21.0, IBM-SPSS, Armonk, NY, USA) and MedCalc (MedCalc, Ostend, Belgium) statistical software packages, and statistical significance level was set at P value < 0.05.

### Ethics statement

The study has been conducted in conjunction with current ethical standards on the use of human tissue. An approval by the IRB was not needed due to the use of cadavers and not live human subjects.

## Results

**Figure 5:**
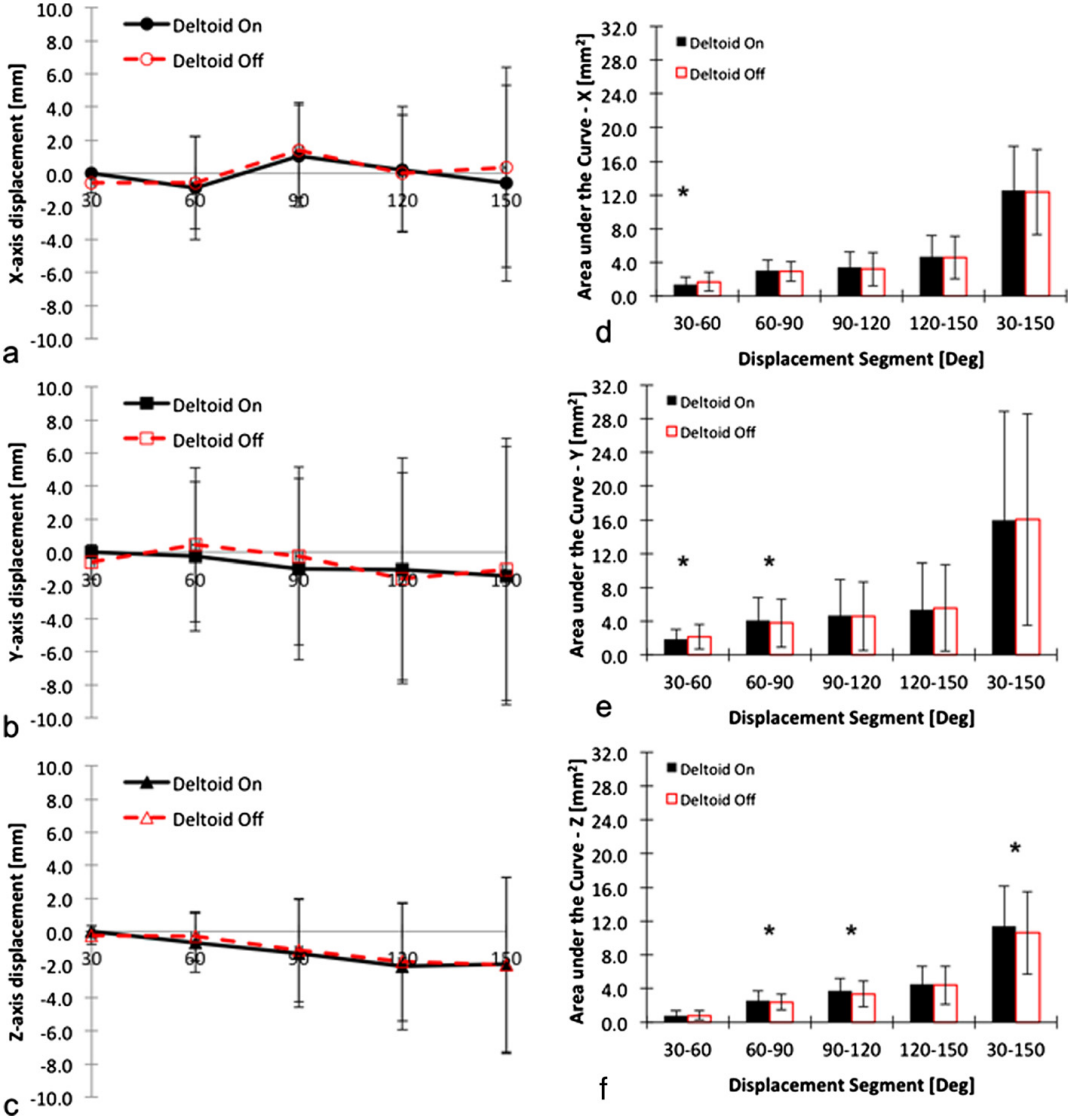
**Presentation of absolute curves per condition for the ABD motion segment in X (a), Y (b) and Z (c) axes, and presentation of AUC curves per condition for ABD motion in X (d), Y (e) and Z (f) axes.** The star (*) highlights significant differences between DON and DOFF (please see text for details). *Legend: ABD = abduction; DON = deltoid intact; DOFF = deltoid removed*.

**Figure 6:**
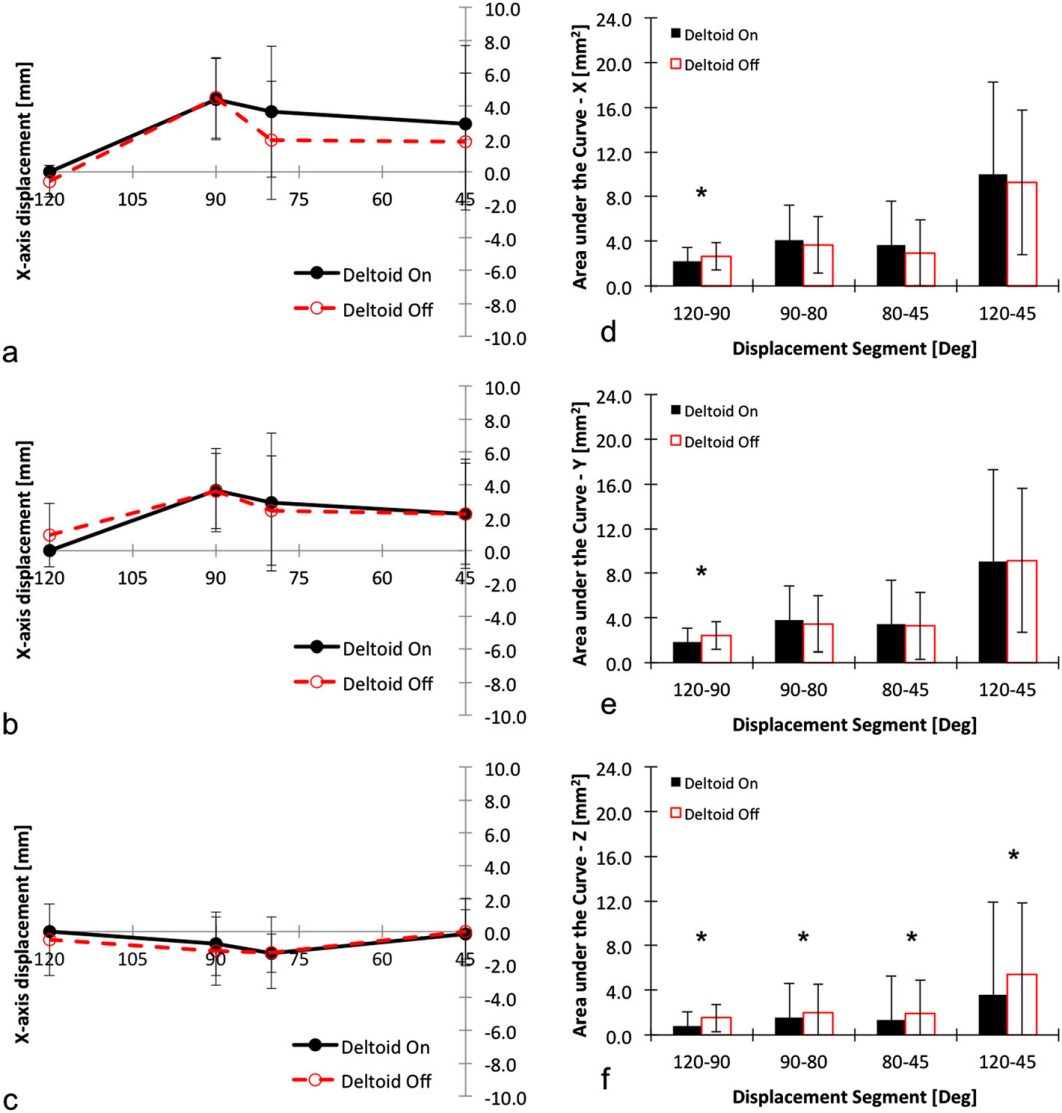
**Presentation of absolute curves per condition for the ATM motion segment in X (a), Y (b) and Z (c) axes, and presentation of AUC curves per condition for ABD motion in X (d), Y (e) and Z (f) axes.** The star (*) highlights significant differences between DON and DOFF (please see text for details). *Legend: AUC = area under the curve; ATM = abbreviated throwing motion; DON = deltoid intact; DOFF = deltoid removed; 120=MAX.*

Statistical analysis revealed that all data was distributed normally in the study (P values greater than 0.05 for all cases). In absolute GH translation, the ABD motion segment (X, Y and Z axes) did not significantly differ between DON and DOFF conditions at the abduction range of ABD^30°^ to ABD^150°^ [Figure 5a, b and c] (Huynh-Feldt within subject effect P values 0.87, 0.88 and 0.92, respectively). Similarly, in the ATM motion segment, the absolute GH translation components (X, Y and Z) did not differ significantly between DON and DOFF conditions at the ATM range of ABER^MAX^ to ABER^45°^ [Figure 6a, b, c] (Huynh-Feldt within subject effect P values 0.62, 0.75 and 0.85 respectively).

There were differences in motion trajectories as revealed by AUC analysis of the two conditions for both the ABD and the ATM motion segments. In case of the ABD segment, there were differences in the X axis (ABD^30°^^−60°^ segment (P < 0.001)), Y axis (ABD^30°^^−60°^ (P = 0.04) and ABD^60°^^−90°^ (P = 0.02)) and Z axis (ABD^60°^^−90°^ (P = 0.01), ABD^90°^^−120°^ (P = 0.04) and ABD^30°^^−150°^ (P = 0.02)) [Figure 5d, e, f]. These results suggest that at 30-60° abduction, the removal of the deltoid resulted in a more superior (X axis) and anterior GH translation (Y axis), while at 60-90° of abduction, the removal of the deltoid resulted in a more inferior (Y axis) and medial displacement of the GH translation (Z axis). From 90° to 120°, the removal of the deltoid resulted in a medialization of the GH translation (Z axis). Overall, the removal of the deltoid resulted in a medialization of the GH translation in abduction (ABD^30°^^−150°^).

In case of the ATM segment, there were differences in X axis (ABER^MAX-90°^ segment (P < 0.001)), Y axis (ABER^MAX-90°^, (P < 0.001) and Z axis (ABER^MAX-90°^, ABER^90°^^−80°^, ABER^80°^^−45°^, ABER^MAX-45°^ (P < 0.01 for all cases)) [Figure 6d, e and f]. These results suggest that from ABER^MAX^ to ABER^90°^ all axes are affected by the removal of the deltoid resulting in a more superior, anterior and more lateral positioning of the glenohumeral translation. However, in the remaining ATM motion segments, only the medio-lateral (Z axis) was involved: the removal of the deltoid resulted in a lateralization of the glenohumeral translation in the ABER^90°^^−80°^and ABER^80°^^−45°^ segments as well as the overall ATM range of motion (ABER^MAX-45°^segment). It thus limits anterior translation in the ABER (abduction-external rotation position).

## Discussion

The purpose of this investigation was to study the passive effect of the deltoid on glenohumeral translations in abduction and abbreviated throwing motion using an intact cadaveric torso. In biomechanical testing, the deltoid is often removed to gain access to the underlying rotator cuff. While this technique is believed to be an acceptable compromise, little data are available to justify this decision, because the passive role of the deltoid has not been described. To improve upon prior descriptions of shoulder kinematics, this investigation employed a novel system designed to evaluate the shoulder using an intact cadaveric torso, rather than an isolated glenohumeral joint.

In this investigation, the removal of the deltoid resulted in significant effects on glenohumeral translation and the center of rotation and thus glenohumeral stability, affirming the importance of the deltoid as a passive restraint with its bulk effect. Its main influence was on the medio-lateral (Z axis) in both motions (ABD and ATM). We could also show a stabilization effect of the deltoid in the segment of ABER^MAX^ to ABER^90°^ as advocated in prior dynamic studies (Halder et al. 2001; Kido et al. 2003).

The effect of shoulder muscles such as the rotator cuff or deltoid muscles on glenohumeral translation has been debated for decades (Markhede et al. 1985; Ovesen and Nielsen 1986; Lee and An 2002; Kido et al. 2003; Colachis et al. 1969; Lee et al. 2000). Since deltoid muscle activity helps to stabilize the glenohumeral articulation, its role as a passive restraint has been attributed to the deltoid’s ability to produce an abduction moment and stabilize the humeral head (Billuart et al. 2007). While describing the ‘bulk effect’ in cadaveric studies, no clear definition of the phenomenon has been developed, even though it is agreed upon that an increase in translation occurs with the removal of tissues that are not directly related to the joint capsule. In an *ex-vivo* study, Ovesen and Nielsen showed increased anterior-posterior glenohumeral translation after removal of the skin and soft-tissues around the shoulder (Ovesen and Nielsen 1986). In another study assessing the effect of muscle volume, the bulk effect was shown when increased muscle volume was correlated with diminished laxity (Howell and Galinat 1988).

Weiner et al. studied plain radiographs showing superior migration of the humeral head (increased superior glenohumeral translation) in static rotator cuff-deficient shoulders, thus supporting the fact of superior pull by the deltoid on the humerus (Weiner and Macnab 1970). An EMG study by Hawkes et al. described the deltoid as a glenohumeral stabilizer in different every-day tasks. The authors studied 13 groups of muscles in healthy subjects including the three parts of the deltoid (anterior, middle and posterior) and studied their activity in a FIT-HaNSA protocol representing different every-day tasks (Hawkes et al. 2012). In an anatomic study, the deltoid functioned to oppose the inferior shear of the latissimus dorsi and the inferior part of the subscapularis muscles (Ackland and Pandy 2009). Moreover, Itoi et al. found that the rotator cuff did not cause a significant bulk effect in a cadaveric study comparing stability at different scapular inclination angles before and after removal. Its removal did not significantly increase glenohumeral translation (Itoi et al. 1993).

Current thinking suggests that scapular positioning might have a much more important role in this motion as described by Itoi et al. (Motzkin et al. 1994; Itoi et al. 1993).

Our model of the throwing shoulder is designed to quantify the passive effects of the shoulder’s supporting soft tissues on glenohumeral motion. Recognizing that the rotator cuff and the glenohumeral ligaments (joint capsule) provide passive stability to the joint at the end range of motion, our cadaveric model does not actively load the joint. For this reason, we have intentionally limited the scope of our investigation to the end range of glenohumeral motion.

The passive effect of the soft tissues is isolated when the humerus is abducted ninety-degrees from the thorax and the humerus is maximally externally rotated. This position coincides with the late cocking/early acceleration phases of the throwing motion, when the throwing athlete’s shoulder experiences the greatest strain, is most vulnerable to injury, and passively stabilized. For the clinical and sports biomechanics community, understanding the forces at this extreme range of motion offers the greatest opportunity for injury prevention, improved rehabilitation, and advancing surgical treatments of shoulder pathology.

As our model does not actively load the glenohumeral joint, we did not evaluate the mid-range of motion, where dynamic muscle contractions are essential for normal glenohumeral mechanics. Flexion and extension are central to the normal range of motion, but do not rely on the passive stability best studied in our model. The results from this study attribute a significant passive role to the deltoid muscle and its dynamic importance in co-contraction and enhancement of the concavity-compression on the glenohumeral joint must also be emphasized (McMahon et al. 2003; Hawkes et al. 2012). The deltoid is a key player in *active* glenohumeral stability (Hawkes et al. 2012; Billuart et al. 2008; Kido et al. 2003; Halder et al. 2001) and appears to additionally add stability to the glenohumeral joint by its mere presence, thus supporting the bulk effect theory.

As with any work, this study has certain limitations. This investigation was designed to test a range of motion, but the technique did not control for the forces applied to the cadaver. For this reason, in applying the same trajectories, native differences in range of motion and other inter-specimen variability were not addressed. Secondly, we used a posterior restraint in order to recreate external rotation, which might alter glenohumeral joint kinematics. This was the only way to recreate sufficient external rotation without horizontal abduction.

An important advantage of this study was the use of whole torsos in order to account for the whole shoulder girdle with its surrounding soft-tissues (muscles, tendons, ligaments, skin). The shoulder girdle was allowed to move freely in all axes and was not restricted by any tension wires.

## Conclusions

Results of this cadaveric study indicate that removal of the deltoid muscle significantly affects the center of rotation for the glenohumeral joint during abduction and abbreviated throwing motion. In this way, the deltoid appears to have an important passive role in lateralization of the GH translation in the pitching motion and its medialization in the GH translation in abduction. Additionally, removal of the deltoid appears to limit anterior translation in the Abduction external rotation position.
